# Nuevo bloqueo interauricular avanzado atípico con morfología trifásica

**DOI:** 10.47487/apcyccv.v6i1.476

**Published:** 2025-02-12

**Authors:** Jorge P. Juárez-Lloclla, Marco A. Lazo-Soldevilla, Sofía Rivera-Chávez, Cengiz Burak, Adrián Baranchuk

**Affiliations:** 1 Hospital de la Amistad Perú-Corea, Santa Rosa II-2, Piura, Perú. Hospital de la Amistad Perú-Corea, Santa Rosa II-2 Piura Perú; 2 Hospital Nacional Ramiro Prialé, Huancayo, Perú. Hospital Nacional Ramiro Prialé Huancayo Perú; 3 Hospital Hidalgo Atoche, Chancay, Perú. Hospital Hidalgo Atoche Chancay Perú; 4 Division of Cardiology, Kingston Health Science Center, Kingston, Ontario, Canada. Division of Cardiology Kingston Health Science Center Kingston, Ontario Canada

*Sr. Editor*

Desde su descripción en el año 1979, el bloqueo interauricular avanzado (BIA) ha cobrado gran interés por lo que múltiples estudios se han desarrollado caracterizando esta entidad. El BIA puede ser típico o atípico sobre la base de los criterios descritos en 2018 por Bayés de Luna *et al.* El típico se caracteriza por una onda P ≥120 ms y una morfología bifásica (+/-) en derivadas inferiores (II, III, aVF). El atípico se clasifica de acuerdo con la morfología (bifásica diferente al BIA típico: tipo I, II, III) y duración de la onda P (<120 ms con morfología de BIA típico) ^1^. Recientemente se agregó un nuevo grupo a la clasificación morfológica del BIA atípico: tipo IV, basado en dos casos que presentaron una morfología trifásica de la onda P en derivadas inferiores nunca descrita ^2^.

Es bien conocida la relación del BIA típico o atípico con arritmias auriculares, principalmente con fibrilación auricular (FA), que cuando está presente se denomina síndrome de Bayés ^3^. La evidencia sigue creciendo y se ha relacionado con un aumento de riesgo de accidente cerebrovascular en estos pacientes, por lo que es indispensable seguir caracterizando este trastorno auricular ^2^^,^^3^.

Presentamos dos nuevos casos con patrón de BIA atípico con morfología trifásica recientemente definida. El primer caso es un varón de 68 años, sin antecedentes conocidos y asintomático cardiovascular que acude para evaluación preoperatoria. El electrocardiograma (ECG) presenta, una onda P inusual con duración de 123 ms, y morfología trifásica en derivadas DII, DIII y aVF (Figura 1 A). En el ecocardiograma se evidenció cardiopatía hipertensiva con función sistólica del ventrículo izquierdo (VI) preservada, hipertrofia moderada de VI, y dilatación leve de aurícula izquierda (AI). En un holter de 24 h se observó contracciones auriculares esporádicas (<200 en 24 h) y una salva auricular. 

**Figura 1:**
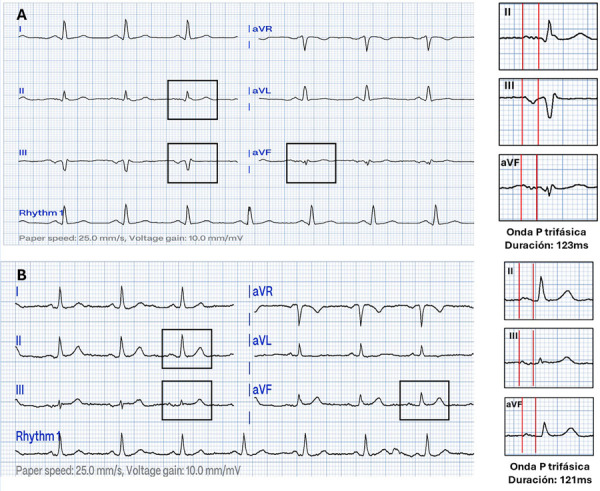
A) Electrocardigorama de caso 1 que muestra una onda P de 123ms de duración y morfología trifásica en DII (+/+/+), DIII (+/-/-) y aVF (+/+/+), compatible con nuevo patrón de bloqueo interauricular atípico de morfología tipo IV. B) Electrocardiograma de caso 2 que muestra una onda P de 121 ms de duración con la misma morfología trifásica del caso A.

El segundo caso se trata de una mujer de 79 años con antecedentes de hipertensión arterial y diabetes *mellitus* controladas, en cuyo ECG presenta una onda P inusual con duración de 121 ms y morfología trifásica en derivadas DII, DIII y aVF (Figura 1 B). En el ecocardiograma se encontró una FEVI del 60%, sin otra alteración.

Recordemos que la activación auricular normalmente se origina en el nódulo sinusal dirigiéndose, con rotación antihoraria, hacia abajo y a la izquierda, lo que origina una onda P positiva en las derivaciones II y aVF, variable en III y aVL, y negativa en aVR. La conducción del estímulo del nódulo sinusal al nódulo auriculoventricular se realiza sin auténticos haces, mientras que la conducción de la aurícula derecha a la izquierda se hace fundamentalmente por la parte alta de la aurícula a través del haz de Bachmann, lo cual permite una conducción más rápida ^2^^,^^4^. El BIA es una expresión del remodelado eléctrico auricular y está relacionado con la disfunción electromecánica de las aurículas; su génesis involucra procesos inflamatorios, infiltrativos, isquémicos y factores degenerativos, principalmente sobre la región de Bachmann ^5^.

A la clasificación descrita en el 2018 del BIA, Silvestrini *et al.* agregaron un nuevo patrón de BIA atípico denominado tipo IV, que muestra una onda P severamente prolongada (onda P ≥160 ms) y morfología trifásica en todas las derivaciones inferiores con P (+/+/-) y P (+/- /+), lo que manifiesta tres momentos de despolarización auricular (despolarización de aurícula derecha, de aurícula izquierda retardada por alteración de la región de Bachmann, y despolarización septal interauricular) ^2^^,^^6^. Los pacientes presentados no cuentan con antecedentes quirúrgicos o de ablación cardiaca, ni disfunción del ventrículo izquierdo, a diferencia de los dos casos reportados inicialmente; pero sí manifiestan comorbilidades y edad avanzada, típicamente relacionados con el BIA ^7^, y también reportado en un último caso presentado de morfología trifásica ^6^. En uno de nuestros pacientes se encontró algunas arritmias auriculares por Holter 24 h. Además, nuestros casos tienen una morfología diferente a los publicados recientemente.

En estos casos, el patrón trifásico de la onda P en las derivaciones DII (+/+/+), DIII (+/-/-) y aVF (+/+/+) podría explicarse por tres vectores de despolarización de las aurículas: 1) la despolarización rápida de la pared lateral de aurícula derecha; 2) el retraso de la despolarización de la aurícula derecha faltante junto con la activación caudo craneal de la aurícula izquierda a través de conexiones musculares alrededor del seno coronario (secundaria a la alteración de la conducción por el haz de Bachmann), y 3) la activación medio cráneo caudal desde conexiones por la fosa ovalis a la vena pulmonar superior derecha y/o del *septum* interauricular.

Se ha constatado la asociación del BIA con el pronóstico en las arritmias supraventriculares (especialmente FA) ^8^, y en distintas situaciones clínicas como el accidente cerebrovascular, deterioro cognitivo y mortalidad ^2^^,^^3^^,^^5^^-^^7^^,^^9^. El BIA puede predecir la recurrencia de FA en pacientes poscardioversión eléctrica ^10^, y también parece conferir un riesgo similar que la FA para el desarrollo de arritmias potencialmente mortales y muerte cardiaca en la miocardiopatía dilatada ^11^. El reconocimiento de los diversos tipos de BIA avanzados junto con los últimos descritos (tipo IV) es de vital importancia pronóstica en los pacientes dentro de múltiples escenarios clínicos, como se ha comentado previamente.

Los nuevos avances en la investigación de la electrofisiología y las imágenes cardiacas continuarán proporcionando más información sobre la importancia de estos nuevos patrones de BIA.

## Financiamiento:

Autofinanciado
